# Incidental Rectal Neuroendocrine Tumor Detected During Screening Colonoscopy Managed With Transanal Minimally Invasive Surgery (TAMIS): A Case Report

**DOI:** 10.7759/cureus.113013

**Published:** 2026-07-20

**Authors:** Rigoberto Rodriguez Ortega, Nora Lis Flores Olmos, Regina De la Garza Torres

**Affiliations:** 1 General Surgery, Hospital General Instituto de Seguridad y Servicios Sociales de los Trabajadores del Estado (ISSSTE) "Dr. Aquiles Calles Ramírez", Tepic, MEX; 2 General Surgery, Hosptial Regional Valentín Gómez Farias, Zapopan, MEX; 3 General Surgery, Universidad Autónoma de Guadalajara, Zapopan, MEX

**Keywords:** incidental finding, neuroendicrine neoplasm, rectal neuroendocrine tumor, screening colonoscopy, transanal minimally invasive surgery (tamis)

## Abstract

Rectal neuroendocrine tumors (NETs) are uncommon neoplasms that are increasingly detected due to the widespread adoption of colorectal cancer screening programs. They typically present as small submucosal nodules, facilitating early diagnosis and minimally invasive treatment. We present the case of a 58-year-old woman with a history of type 2 diabetes mellitus who underwent screening colonoscopy while asymptomatic. During the procedure, an 18-mm non-polypoid submucosal lesion was identified on the anterior rectal wall, 8 cm from the anal verge. Contrast-enhanced computed tomography of the chest, abdomen, and pelvis demonstrated no evidence of regional or distant metastasis.

Complete local excision was performed using transanal minimally invasive surgery (TAMIS) without complications. Histopathological examination revealed a well-differentiated grade 1 rectal NET staged as pT1bN0M0, with a Ki-67 proliferation index of 1%, positive immunohistochemical staining for chromogranin A, negative surgical margins, and no evidence of lymphovascular or perineural invasion. Follow-up imaging demonstrated no evidence of local recurrence or distant metastatic disease, and the patient remains under clinical and radiological surveillance. This case highlights the importance of colorectal cancer screening programs for the incidental detection of rare rectal NETs and supports TAMIS as a safe and effective treatment option for selected patients with localized disease.

## Introduction

Rectal neuroendocrine tumors (NETs) are uncommon neoplasms arising from enteroendocrine cells of the diffuse neuroendocrine system. Their detection has increased with the broader use of colorectal cancer screening and improvements in endoscopic imaging. Most rectal NETs are small, well-differentiated, and clinically silent; consequently, they are frequently discovered incidentally during colonoscopy performed in otherwise asymptomatic patients [1].

Management is guided by a combination of tumor size, histological grade, depth of invasion, lymphovascular involvement, proliferative activity, and regional or distant spread. Small, superficial tumors can often be treated endoscopically, and modified endoscopic mucosal resection techniques have demonstrated high rates of complete resection in appropriately selected lesions [2]. However, management becomes less straightforward for tumors measuring between 10 and 20 mm, in which the risk of incomplete excision or metastatic behavior must be balanced against the morbidity of radical surgery [3]. When the initial endoscopic resection is incomplete, residual tumor may be detected during salvage treatment. However, the impact of routine additional intervention on recurrence remains uncertain; therefore, treatment decisions should be individualized [4].

For localized lesions that require full-thickness excision or more reliable margin control, transanal minimally invasive surgery (TAMIS) offers an organ-preserving alternative to radical rectal surgery. Contemporary evidence supports transanal minimally invasive approaches as technically feasible procedures that can achieve adequate local excision while limiting perioperative morbidity and preserving anorectal function [5]. We present the case of an incidentally detected, well-differentiated grade 1 rectal NET successfully managed with TAMIS, highlighting the importance of screening, careful pathological risk assessment, and individualized selection of local therapy.

## Case presentation

**Figure 1:**
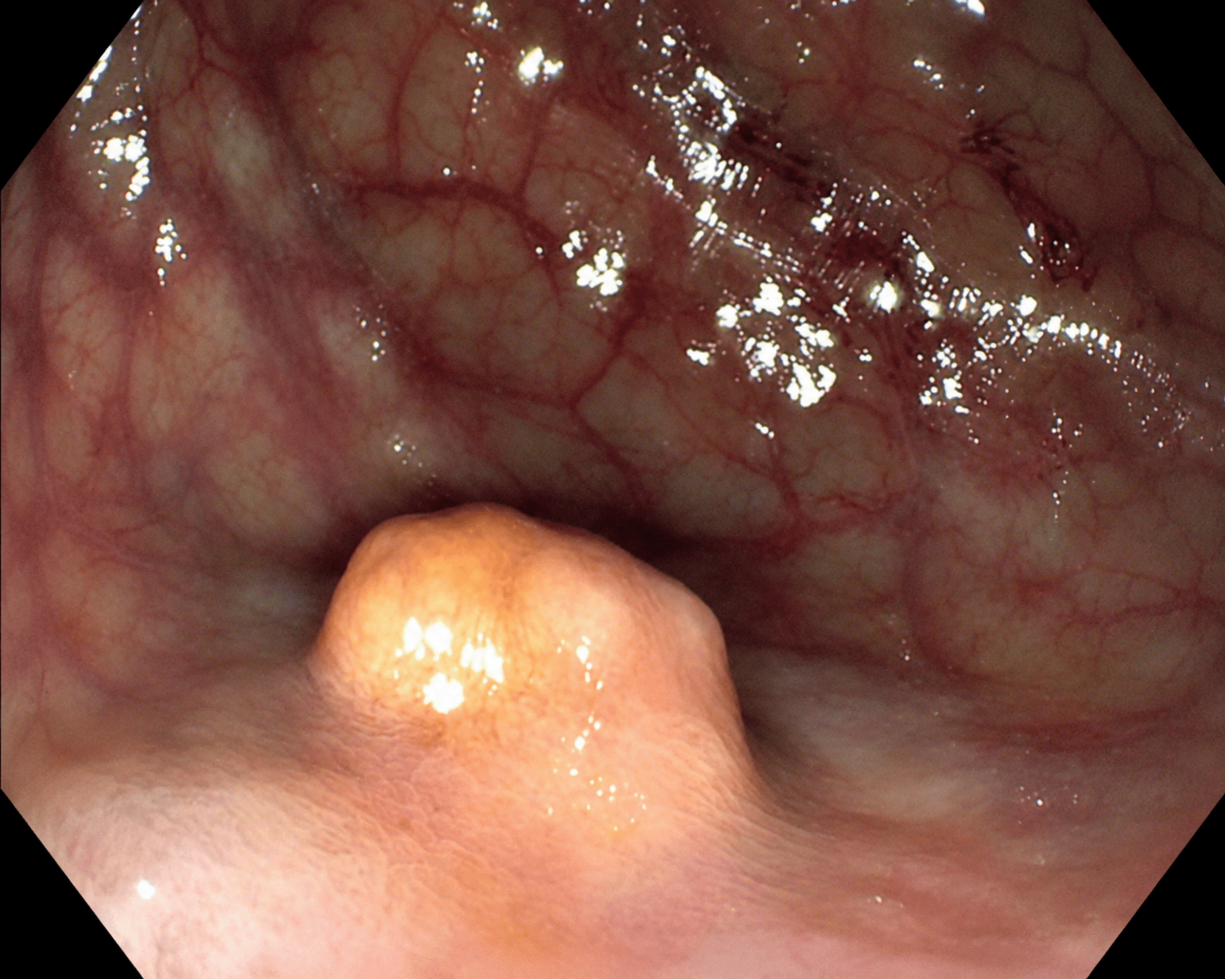
Colonoscopic view of a rectal neuroendocrine tumor located 8 cm from the anal verge.

In August 2024, a 58-year-old woman with an eight-year history of type 2 diabetes mellitus, with no history of tobacco or alcohol use and no family history of colorectal cancer, underwent screening colonoscopy at Hospital General Instituto de Seguridad y Servicios Sociales de los Trabajadores del Estado (ISSSTE) "Dr. Aquiles Calles Ramírez", Tepic, Nayarit, Mexico. She was asymptomatic at presentation. Colonoscopic examination identified a solitary 18-mm non-polypoid submucosal lesion located on the anterior wall of the rectum, 8 cm from the anal verge (Figure 1).

**Figure 2:**
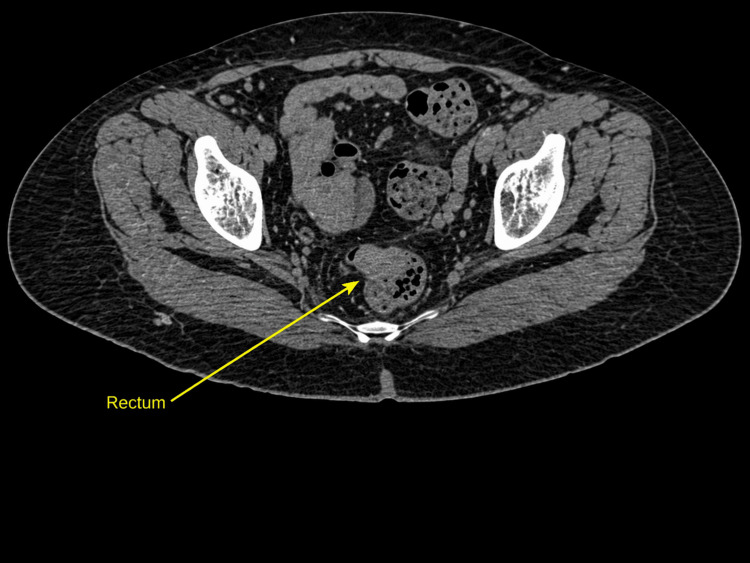
Contrast-enhanced axial pelvic computed tomography demonstrating the rectum (arrow) with no radiological evidence of mesorectal lymphadenopathy, locoregional invasion, or distant metastatic disease, consistent with localized disease at presentation.

Staging evaluation with contrast-enhanced computed tomography of the chest, abdomen, and pelvis demonstrated no evidence of locoregional involvement or distant metastatic disease (Figure 2).

**Figure 3:**
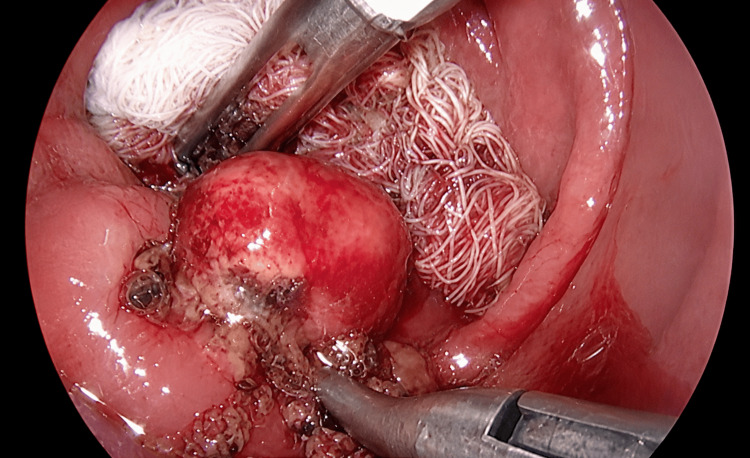
Transanal minimally invasive surgery dissection of a 1 × 1 cm rectal neuroendocrine tumor.

Complete local excision was performed using TAMIS. The operative time was 60 minutes, with an estimated blood loss of 10 mL. The procedure was completed without intraoperative or postoperative complications, and the patient was discharged one day after hospitalization (Figure 3). 

**Figure 4:**
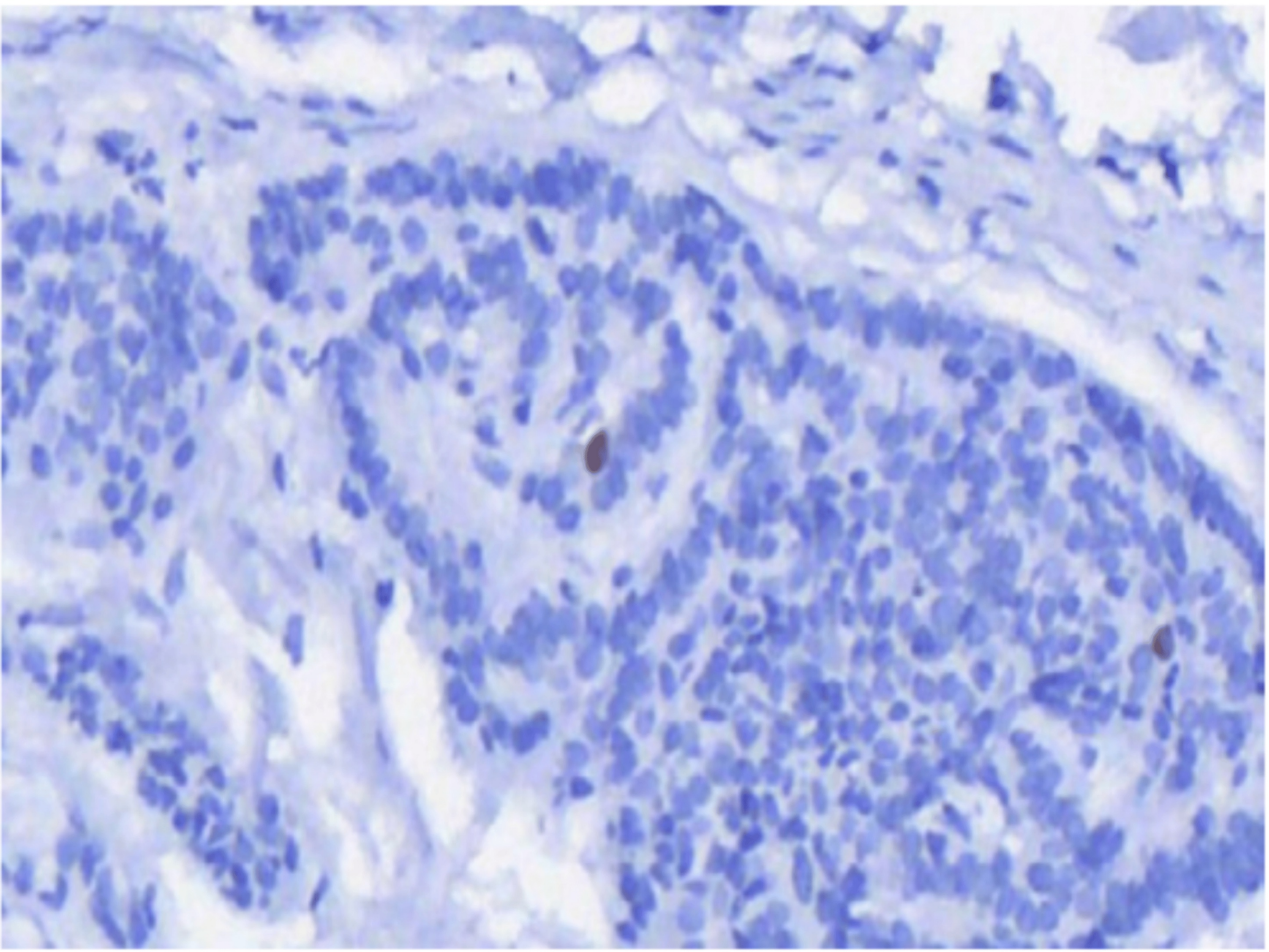
Immunohistochemical analysis of the rectal neuroendocrine tumor demonstrating positive chromogranin A staining and a low Ki-67 proliferation index (1%), consistent with a well-differentiated grade 1 neuroendocrine tumor.

Histopathological examination revealed a well-differentiated grade 1 rectal NET with positive immunohistochemical staining for chromogranin A and a Ki-67 proliferation index of 1%. Histopathological evaluation confirmed negative surgical margins (R0 resection), with no evidence of lymphovascular or perineural invasion. The corresponding serum chromogranin A level was 23.3 ng/mL (Figure 4).

Clinical and radiological follow-up at 12 months demonstrated no evidence of local recurrence or distant metastatic disease.

## Discussion

Rectal NETs originate from enteroendocrine cells of the diffuse neuroendocrine system and represent the most common gastrointestinal neuroendocrine neoplasms identified in the rectum. Their increasing detection has been attributed to the widespread implementation of colorectal cancer screening programs and the greater use of high-definition endoscopy [6,7]. In the present case, the tumor was detected incidentally during screening colonoscopy in an asymptomatic patient, illustrating the important role of colorectal cancer screening in the early diagnosis of these lesions.

Endoscopically, rectal NETs typically appear as small submucosal lesions with a smooth surface and yellowish coloration, features that may mimic benign lesions and contribute to incomplete resection if their neuroendocrine origin is not recognized [8]. Our patient presented with these characteristic endoscopic findings, which prompted complete diagnostic evaluation and definitive surgical management.

The frequent detection of rectal NETs at an early stage is closely related to the growing use of screening colonoscopy, allowing diagnosis before the development of advanced disease [9]. This was reflected in our patient, whose tumor was diagnosed before the onset of symptoms or metastatic spread.

The biological behavior of rectal NETs depends mainly on tumor size, histological grade, proliferative activity, and depth of invasion. Increased Ki-67 expression and invasion beyond the submucosa have been associated with a higher risk of lymph node involvement and distant metastasis, even in relatively small lesions [10,11]. In our case, the tumor demonstrated a Ki-67 proliferation index of 1%, no lymphovascular or perineural invasion, and negative surgical margins, findings consistent with a favorable prognosis.

Most rectal NETs remain asymptomatic and are diagnosed incidentally during screening colonoscopy. When symptoms occur, they are usually nonspecific and may include rectal bleeding, altered bowel habits, or tenesmus. Functional hormonal syndromes are uncommon because these tumors generally exhibit limited hormonal activity, and the secreted substances undergo extensive hepatic metabolism before reaching the systemic circulation [12,13,14]. Consistent with previous reports, our patient remained completely asymptomatic before diagnosis.

Histopathological examination with immunohistochemical staining for chromogranin A and synaptophysin remains essential for definitive diagnosis and prognostic assessment [15]. In our patient, immunohistochemistry confirmed the diagnosis, supporting the pathological findings and final staging.

## Conclusions

Rectal NETs are increasingly detected through colorectal cancer screening, allowing diagnosis at earlier stages and facilitating organ-preserving treatment. In the present case, an incidentally detected localized grade 1 rectal NET was successfully treated with TAMIS, achieving complete resection with negative surgical margins and no evidence of recurrence after 12 months of follow-up. This case highlights the importance of individualized management based on tumor characteristics and supports TAMIS as a safe and effective treatment option for selected localized rectal NETs.
